# Low genetic diversity in a critically endangered primate: shallow evolutionary history or recent population bottleneck?

**DOI:** 10.1186/s12862-019-1451-y

**Published:** 2019-06-26

**Authors:** Weiran Wang, Yitao Zheng, Jindong Zhao, Meng Yao

**Affiliations:** 1 School of Life Sciences, Peking University, Beijing, 100871 China; 2 Institute of Ecology, Peking University, Beijing, 100871 China; 3 Beijing National Day School, Beijing, 100871 China

**Keywords:** Approximate Bayesian computation, Bottleneck effect, Genetic variation, Population demography, White-headed langur, *Trachypithecus leucocephalus*

## Abstract

**Background:**

Current patterns of population genetic variation may have been shaped by long-term evolutionary history and contemporary demographic processes. Understanding the underlying mechanisms that yield those patterns is crucial for informed conservation of endangered species. The critically endangered white-headed langur, *Trachypithecus leucocephalus*, is endemic to a narrow range in southwest China. This species shows very low genetic diversity in its 2 main relict populations, Fusui and Chongzuo. Whether this has been caused by a short evolutionary history or recent population declines is unknown. Therefore, we investigated the contributions of historical and recent population demographic changes to population genetic diversity by using 15 nuclear microsatellite markers and mitochondrial DNA (mtDNA) control region sequences.

**Results:**

Using genetic data from 214 individuals we found a total of 9 mtDNA haplotypes in the Fusui population but only 1 haplotype in the Chongzuo population, and we found an overall low genetic diversity (haplotype and nucleotide diversities: *h* = 0.486 ± 0.036; *π* = 0.0028 ± 0.0003). The demographic history inferred from mtDNA and microsatellite markers revealed no evidence for historical population size fluctuations or recent population bottlenecks. Simulations of possible population divergence histories inferred by DIYABC analysis supported a recent divergence of the Chongzuo population from the Fusui population and no population bottlenecks.

**Conclusions:**

Despite severe population declines caused by anthropogenic activities in the last century, the low genetic diversity of the extant white-headed langur populations is most likely primarily due to the species’ shallow evolutionary history and to a recent, local population founder event.

**Electronic supplementary material:**

The online version of this article (10.1186/s12862-019-1451-y) contains supplementary material, which is available to authorized users.

## Background

Genetic diversity describes the differences in DNA sequences among individuals of a population or of a species and is a key aspect of biological evolution [1]. Genetic variation or polymorphism contributes to the viability and evolutionary potential of natural populations and has important implications in the conservation of endangered species [2]. For instance, low levels of genetic diversity in many plant and animal species have been linked to reduced reproductive success, increased susceptibility to infectious diseases, and impaired adaptability to environmental challenges [3, 4]. Therefore, a central task for ecologists and conservation biologists is to accurately assess current levels and distributions of genetic diversity in natural populations [5, 6].

Many natural and anthropogenic factors at various temporal and spatial scales, including evolutionary history and recent human-related population declines, can influence current genetic variation in a species, thus figuring prominently in determining population genetic patterns. For example, late speciation or population vicariance events cause lower genetic variance, compared to populations with deep divergence histories [7, 8], since new genetic polymorphisms take generations to emerge and accumulate in a population. On the other hand, populations that have suffered recent, severe declines in size due to anthropogenic causes (e.g., habitat destruction/fragmentation, over exploitation, introduction of invasive species) often show depletion of genetic diversity due to the loss of genetic polymorphism suffered during such population bottlenecks, as well as further loss of variation through genetic drift, post-bottleneck [9, 10]. Accurate assessment of underlying mechanisms that determine current population genetic patterns is crucial for both understanding the evolutionary history of and uncovering contemporary threats to endangered species, and both are prerequisites for effective conservation and management design [11]. Molecular tools using genetic markers with different mutation rates and inheritance characteristics can help disentangle population demographic histories of various timescales. For instance, relatively slow mutation rates of mitochondrial DNA (mtDNA) sequences allow for analysis of ancient evolutionary events and population history [12, 13], whereas rapidly mutating microsatellite markers can provide information on recent population genetic processes [14, 15].

Asian colobines are a unique clade of primates including 50–60 species known for their highly diverse morphological characteristics and ecological adaptations [16, 17]. Mostly found in various forest types of South and Southeast Asia, these species often live in extremely restricted ranges with increasingly fragmented habitat and they are facing serious threats of local extirpation or even range-wide extinction [18]. Despite their serious endangered statuses, only a few species have had their evolutionary histories, genetic diversities, and population structures assessed (e.g., snub-nosed monkeys, [19, 20]), and those data are lacking for the majority of Asian colobines.

**Fig. 1:**
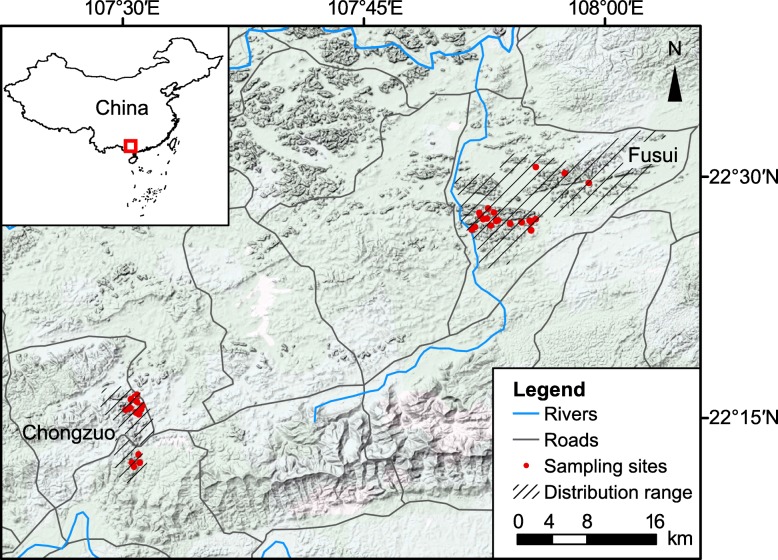
Map (Map data: Google, DigitalGlobe) of the study area showing sites of sampled white-headed langur groups

The white-headed langur (WHL), *Trachypithecus leucocephalus*, is a lesser known Asian colobine monkey living in a very narrow range (current total distribution area is about 80 km^2^) in southwestern China [21]. Its suitable habitats, forested karst limestone hills, occur amidst agricultural and rural residential lands and are highly fragmented by human activities [22]. In the last century, this species has suffered severe population declines, mostly due to habitat modification and fragmentation and to hunting, with an estimated 80% contraction of its total range and a 60% population size reduction, including local extirpation in some regions [21, 23, 24]. With approximately 1000 individuals remaining in the wild, the WHL is recognized as a critically endangered species by the IUCN Red List [25]. Currently, over 90% of WHL populations reside in 2 counties, Fusui (FS) and Chongzuo (CZ), that are separated by about 50 km of non-habitable, flat land [26] (Fig. 1). Each area is further fragmented to various degrees by farmlands and human residences [22]. Previous analyses using microsatellite data indicated an overall low genetic diversity and significant differentiation between the FS and CZ populations [22, 28]. Mitochondrial DNA control region sequences from 77 individuals revealed low diversity in the FS population and a complete lack of haplotype diversity in the CZ population [28]. Lacking historical demographic and genetic data, how such patterns of population genetic variation arose remains unclear.

In this study, we evaluated WHL population genetic diversity using new mtDNA sequence data obtained from most of its range, and then generated the first molecular-based inferences of the historical and recent population demographics of the WHL using a combined dataset of mtDNA and microsatellite genotypes. We aimed to answer 2 questions: (i) Is the current range-wide, low, population genetic diversity the result of a short evolutionary history or of recent population declines; and (ii) Is the extremely low genetic diversity in the CZ population attributable to a recent founder event or to severe population bottlenecks? In light of our findings, we discuss the evolutionary origin and colonization history of the WHL and provide conservation planning suggestions that can help enhance population viability.

### Sampling and fecal DNA analyses

Between 2012 and 2014 we non-invasively collected fecal samples from wild WHL populations in FS and CZ in the Chongzuo National White-Headed Langur Nature Reserve (22°10′43″–22°36′55″ N, 107°16′53″–107°59′46″ E) in Guangxi Province, China. We collected a total of 403 fecal samples (FS: 245, CZ: 158) from 41 wild WHL reproductive groups, with 1–26 (mean = 9.8) samples collected per group. Details of sampling were described previously [22, 28]. The sampling areas covered the distribution range of 90% of the extant WHL population and our samples represented one-third of all known reproductive groups. Details of fecal DNA extraction and the molecular analyses of 15 nuclear microsatellite markers, the mtDNA hypervariable region I (HVRI), and a sex identification marker have been described in previous studies [22, 28, 37]. (See also Additional file 1: Table S1 and Table S2 for primer information.) Polymorphic microsatellite markers were specifically developed for this species [37]. We identified 214 unique multi-locus genotypes (FS: 131, CZ: 83) among the samples and regarded each as an independent individual in our genetic analyses. These individuals were from 39 reproductive groups (FS: 21, CZ: 18), with 1–18 individuals (mean = 5.5) per group [27]. This microsatellite dataset has been used in previously published studies of genetic diversity, population structure, and dispersal patterns [22, 38]. We used the same microsatellite dataset for analyses of population demographics in this study.

A previous study found that all the variable sites of a 1213-bp sequence of the mtDNA control region reside within a fragment of 350-bp in the HVRI of the WHL [28]. This study’s 350-bp HVRI fragment was comparable to sequences used in several other population genetic studies of primates [39–41]. We successfully amplified and sequenced the HVRI fragment for all but 1 of the 214 unique individuals identified using microsatellite data. A subset of the HVRI sequences from 77 individuals had been used in previous analyses of genetic diversity and demographic history [28], and sequences from the other 136 individuals were first reported in this current study. HVRI sequences from all 213 individuals were used for mtDNA analyses in this study.

### Genetic diversity and population structure inferred from mtDNA data

By using microsatellite and mtDNA sequence data with multiple analytical approaches of population structure, previous studies showed both strong genetic differentiation between the FS and CZ populations and limited within-population divergence [22, 28]. Although the genetic structure between 2 habitat patches within the FS population was also suggested by some analyses [22], the limited geographic distance (< 3 km at the widest part) and small genetic differentiation do not support a long-time separation of the 2 patches. Because the main goal of this study was to resolve the population demographic history, we regarded FS and CZ as 2 distinct populations, without considering intra-population genetic structure in our analyses.

We aligned and edited the mtDNA sequences with CLUSTAL X v 2.1 [42]. Genetic diversity parameters, including the numbers of polymorphic sites (*s*) and haplotypes (*n*), haplotype diversity (*h*), and nucleotide diversity (*π*) [43] of the sequences, were estimated for each population using DNASP v 5.10.01 [44]. The Hasigawa-Kishino-Yano (HKY) model of nucleotide substitution was the most appropriate model, as indicated by the corrected Akaike Information Criterion [45] and the hierarchical likelihood-ratio tests in MODELGENERATOR [46]. Therefore, this model, or the most similar available model, was used in subsequent analyses. Then, using NETWORK v 4.6.1.2 [47], we constructed a median-joining network of HVRI haplotypes to show their mutational relationships and geographical distributions.

To estimate genetic differences among populations, we used mtDNA data in ARLEQUIN v 3.5.1.2 [48, 49] to calculate pairwise *Φ*_ST_ [50] and *F*_ST_ [51] statistics between the populations. The significance of pairwise comparisons was tested with 10,000 permutations.

### Demographic history inferred from mtDNA data

Historical changes in the WHL population size were inferred from mtDNA data by using several methods. First, we performed neutrality tests, including Tajima’s *D* [52] and Fu’s *F*_S_ tests [53], in ARLEQUIN with 10,000 permutations. Second, we inferred demographic changes using mismatch distributions [54] in DNASP with 10,000 bootstrap replicates. Populations experiencing recent growth are expected to show a unimodal and smooth distribution of pairwise differences between mtDNA sequences. We determined the raggedness index (RI) and statistical significance between the observed and expected values under a constant population size model. Finally, to estimate changes in the size of WHL population over time, we conducted Bayesian skyline plot (BSP) analyses [55], implemented in BEAST v 1.8.1 [56]. The analyses were run using the HKY substitution model, a piecewise-constant Bayesian Skyline prior, a strict clock model, and a standard substitution rate of 0.164 per nucleotide per million years in humans and apes [57]. We ran MCMC simulations of 2 × 10^8^ steps for the total population (FS and CZ combined) and 1 × 10^8^ steps for the FS population, sampling every 1000 steps and discarding the first 10% as burn-in. The analysis was repeated 3 times, each with different seed numbers. The log and tree files of different runs were combined using LOGCOMBINER v 1.8.1. Effective sample size, mixing, and convergence were checked using TRACER v 1.5 [58].

### Population bottleneck inferred from microsatellite data

For the microsatellite data, we used BOTTLENECK v 1.2.02 [59] to assess recent bottleneck events signalled by excessive heterozygosity with respect to expectations under 2 microsatellite mutational models: (i) the stepwise mutational model (SMM) and (ii) the two-phased stepwise mutational model (TPM) with 95% single-step mutations. We evaluated the significance of the tests using sign tests and Wilcoxon’s signed-rank tests, the latter being the most appropriate test when fewer than 20 microsatellite loci are used [59]. We also used Mode-shift distortion tests to detect whether the allele frequency distribution was L-shaped, a typical characteristic of populations that have not experienced recent bottlenecks [60].

### Population divergence history inferred using approximate Bayesian computation and microsatellite data

**Fig. 2:**
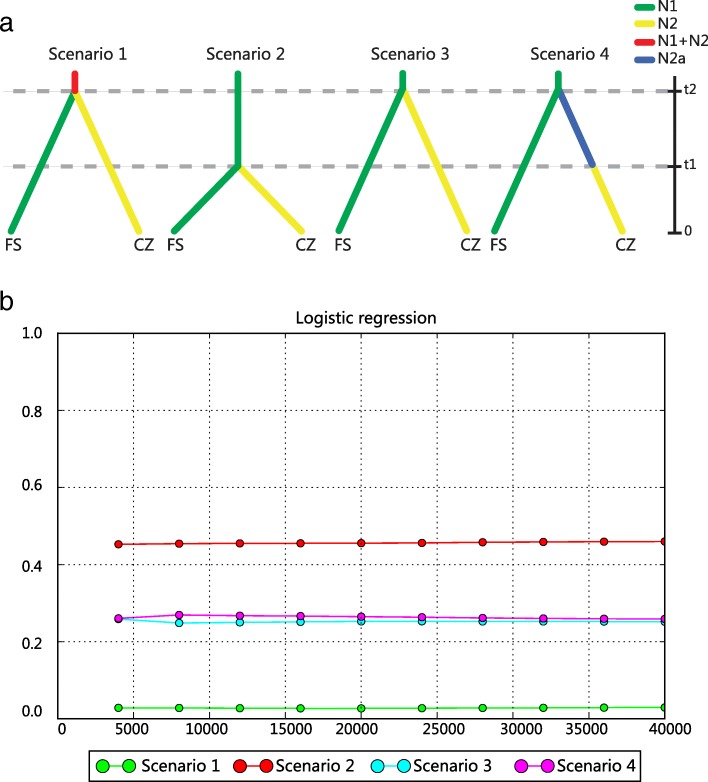
Possible demographic history scenarios for the Fusui (FS) and Chongzuo (CZ) white-headed langur populations. **a** 4 scenarios of population demographic history defined after using DIYABC and microsatellite data include FS and CZ splitting at the same time from a common ancestor (null mode, Scenario l); CZ diverging from FS recently (Scenario 2); CZ diverging from FS at an earlier (historical) time (Scenario 3); and CZ diverging from FS early and then experiencing a recent bottleneck event (Scenario 4). Different colors indicate different populations or sizes: N1, FS population size; N2, CZ population size after divergence or after the proposed bottleneck; N2a, CZ population size before the proposed bottleneck. Time (t1, recent; t2, historical) is not strictly to scale. **b** Logistic regression plot for the simulated scenarios in (a). The *x*-axis indicates the number of simulations used to calculate the probabilities and the *y*-axis indicates the posterior probabilities of each scenario

We used microsatellite data with the approximate Bayesian computation (ABC) method, implemented in DIYABC v 2.0.1 [61, 62], to analyze the population demographic history. Based on observed patterns of mtDNA and microsatellite diversities in the FS and CZ populations (see Results), we speculated that the CZ population had likely originated from the FS population and that the very low genetic variability in the CZ population was due either to recent establishment by a small number of founders from the FS population or to a severe population bottleneck in the last century. Therefore, we defined 4 demographic scenarios for the FS and CZ populations (Fig. 2a). In scenario 1 (null model), FS and CZ split simultaneously from a common ancestral population at historical time *t*_2_. Scenarios 2 to 4 all considered FS as the ancestral population but the demographic history of CZ varied. Scenario 2 considered CZ diverging from FS at a recent time *t*_1_ (*t*_1_ < *t*_2_); scenario 3 considered CZ diverging from FS at an earlier (historical) time *t*_2_; and scenario 4 had CZ first diverging from FS at a historical time *t*_2_, followed by a population bottleneck event in CZ at a recent time *t*_1_. *N*_2a_ and *N*_2_ were effective population sizes of the CZ population before and after the proposed population bottleneck, respectively (*N*_2_ < *N*_2a_) (Fig. 2a). Because of the significant, inferred genetic differentiation between the FS and CZ populations, based on both microsatellite and mtDNA data ([38]; this study), we did not consider gene flow between populations in our simulations. We set prior values for the effective population size and divergence time estimates with a uniform distribution for all parameters (Additional file 1: Table S3). In all scenarios, except for the CZ population in scenario 4, we assumed that each population maintained a constant size over time, post-divergence from the ancestral population. The microsatellite mutation rate was set between 1 × 10^− 5^ and 1 × 10^− 3^ substitutions/generation with a uniform distribution and under the stepwise mutation model. We set the repeat units for the microsatellite loci according to the loci information [37] and used default settings for the other microsatellite parameters. We simulated one million datasets for each scenario and calculated summary statistics (mean number of alleles per locus, mean genetic diversity, and mean Garza-Williamson’s *M*) for each population and *F*_ST_ and the mean classification index between pairs of populations. Posterior probabilities of the modeled scenarios were estimated using a logistic regression approach [62] with the 1% of simulated datasets possessing the smallest Euclidian distances to the observed dataset. Goodness-of-fit between the simulated and real datasets was evaluated using principal component analysis (PCA) in DIYABC.

## Results

### Genetic diversity and population structure inferred from mtDNA data

**Fig. 3:**
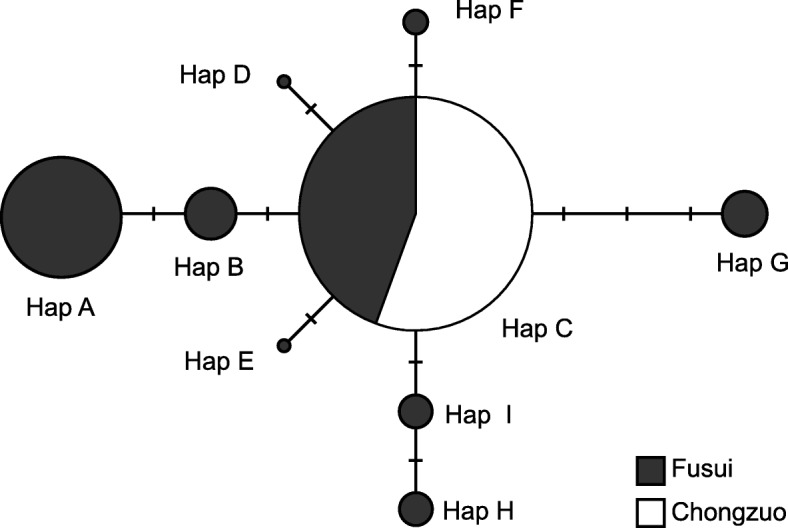
Median-joining network using mtDNA HVRI haplotypes in the white-headed langur Fusui and Chongzuo populations. The sizes of the circles represent the haplotype frequencies. Each bar on a line connecting 2 haplotypes indicates one mutational step

Of the 403 field-collected fecal samples, 390 (97%) successfully generated sequences of the mtDNA HVRI, corresponding to 213 (FS: 131, CZ: 82) of the 214 distinct individuals previously identified from microsatellite genotypes. We found 9 haplotypes (Hap A–I) consisting of 10 polymorphic sites among all samples (GenBank acc. Nos. KP772243–KP772251; Additional file 1: Table S4). The number of site differences between 2 haplotypes varied from 1 to 5. Each sample exhibited a single haplotype, except for 5 samples from 1 social group in the FS population in which each sample showed 2 haplotypes (Hap H and Hap I). The neighbour-joining network of the mtDNA haplotypes showed a single haplogroup with all other haplotypes differing from Hap C at 1 to 3 nucleotides (Fig. 3).

The total population had low haplotype diversity (*h*) and low nucleotide diversity (*π*) (*h* = 0.486 ± 0.036; *π* = 0.0028 ± 0.0003) (Additional file 1: Table S5) and the haplotype distribution showed a strong geographical pattern, as described earlier. The FS population had all 9 haplotypes while the CZ population had only 1, which was also the most common haplotype (Hap C) in the FS population. While the FS population had low haplotype diversity (*h*) and low nucleotide diversity (*π*) (*h* = 0.653 ± 0.030; *π* = 0.0040 ± 0.0003), the CZ population completely lacked diversity. Within the FS reproductive groups with more than 1 sample (*N* = 20), 7 groups had 1 haplotype, 10 had 2 haplotypes, and 3 had 3 haplotypes. Pairwise *F*_ST_ values were large and statistically significant between the FS and CZ populations (*F*_ST_ = 0.304, *P* < 0.001; *Φ*_ST_ = 0.217, *P* < 0.001), indicating strong genetic differentiation between the 2 populations.

### Demographic history inferred from mtDNA data

**Fig. 4:**
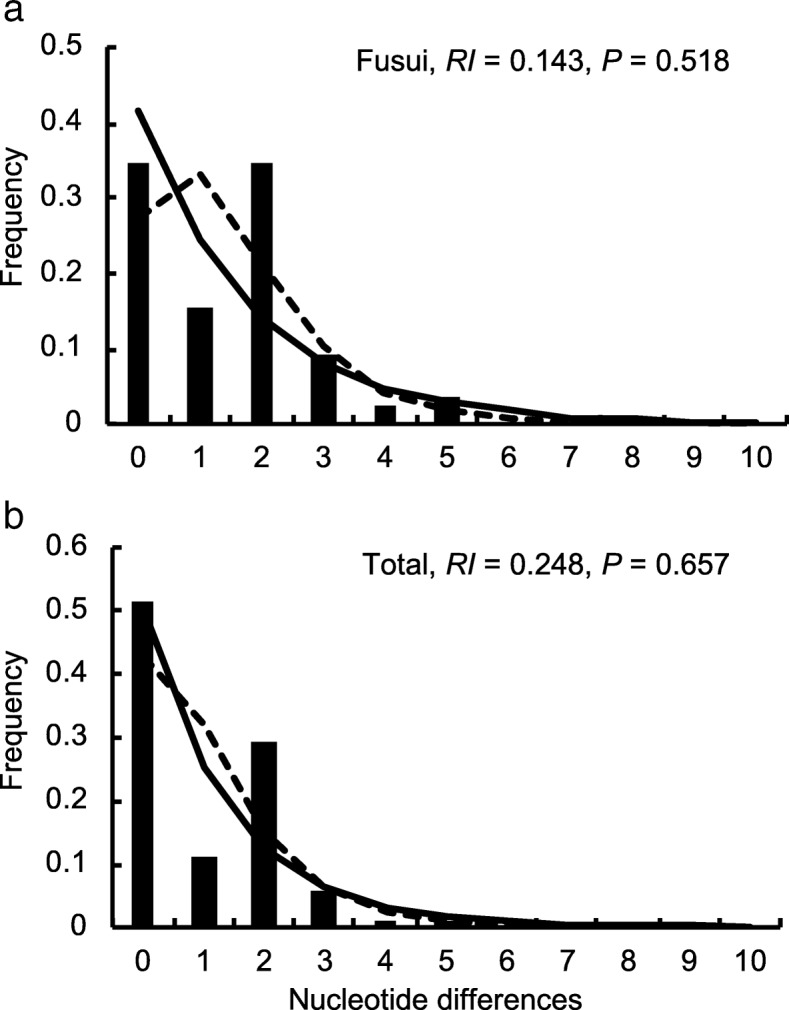
Mismatch distributions based on mtDNA HVRI sequences of the white-headed langur (**a**) Fusui and (**b**) the total (Fusui and Chongzuo combined) populations. The *x*-axis indicates the number of pairwise differences and the *y*-axis indicates the relative frequencies of pairwise comparisons. Observed (solid bars) and expected pairwise differences under the constant population size model (solid line) and the population growth-decline model (dotted line) are shown. Harpending’s raggedness indices (*RI*) and associated *P* values are shown

**Fig. 5:**
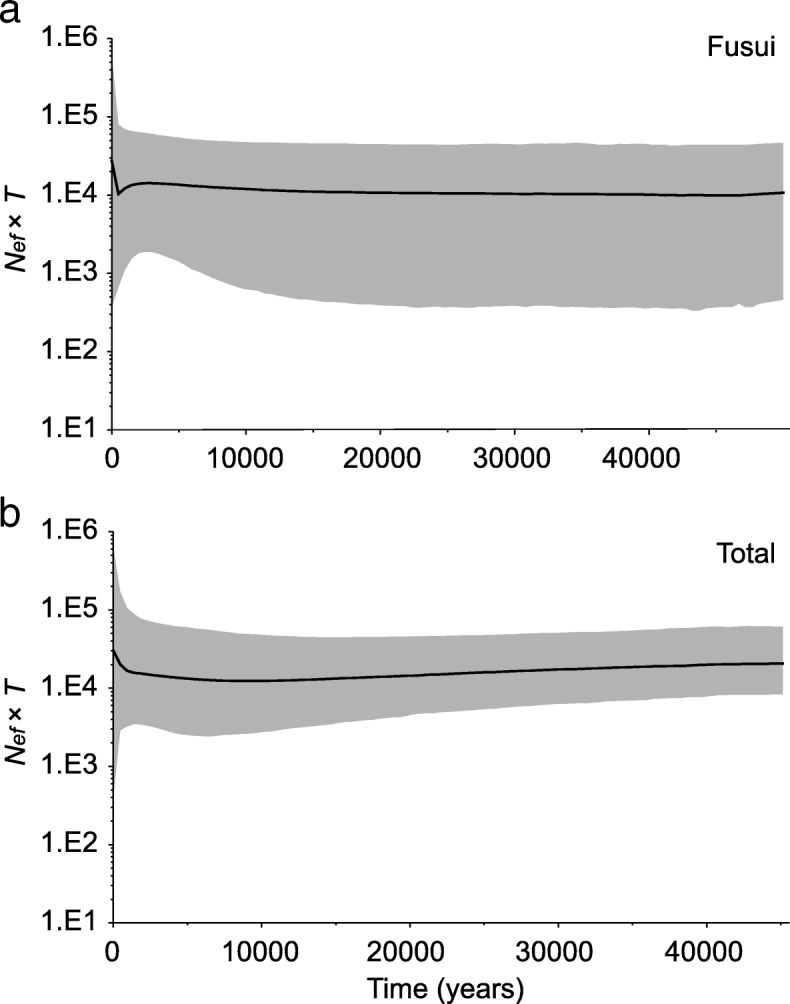
Bayesian Skyline plots based on white-headed langur mtDNA HVRI sequences. **a** Fusui population. **b** total (Fusui and Chongzuo combined) population. The *x*-axis is in years from present (0) to the upper limit of the 95% highest posterior densities (HPD) and the *y*-axis is on a logarithmic scale and in units of the product of female effective population size (*Nef*) and generation time (*t*). The solid lines show the median estimates and the shaded areas represent the 95% HPD

Because the CZ population lacked mtDNA haplotype variation, it was excluded as a population from mtDNA-based demographic analyses. Neutrality tests, including Tajima’s *D* and Fu’s *F*_S_ tests, yielded nonsignificant results in both the FS and the total (FS and CZ combined) population (Additional file 1: Table S5), thus suggesting historical demographic stability. The ragged and multimodal distribution that mismatch analysis yielded also suggested no population expansion in either the FS or the total population (Fig. 4). The demographic history recovered in the BSP agreed with these analyses, displaying an overall stable effective population size over the past 50,000 years (Fig. 5). Taken together, the mtDNA data supported a relatively constant historical population size with no large expansions or contractions.

### Population bottleneck inferred from microsatellite data

BOTTLENECK analyses, using the sign test and the Wilcoxon signed-rank test, found no significant evidence for recent declines in effective population sizes in the FS, CZ, and total populations under the SMM and TPM microsatellite mutation models (*P* > 0.05 for all tests; Additional file 1: Table S6). Mode-shift tests showed L-shaped distributions for all populations, indicating an absence of population bottlenecks.

### Population divergence history inferred using ABC and microsatellite data

The most supported scenario of population demographic history evaluated using DIYABC analysis was scenario 2 (Fig. 2b; see also Additional file 1: Figure S1 for the PCA test result), in which the CZ population split from the FS population recently, with both CZ and FS populations maintaining a constant population size (logistic approach PP = 0.46). The posterior probability of scenario 2 was significantly higher than that of the other scenarios, which varied between 0.03 and 0.26 (Additional file 1: Table S7). The posterior distributions of effective population sizes for the FS and CZ populations had median values of 1980 and 1140, respectively, with the 2 populations diverging about 3050 years ago, given a 10-year generation time (Additional file 1: Table S8, Figure S2).

## Discussion

### Low mtDNA genetic diversity in the WHL

Our results confirmed previous findings, which had used fewer samples than the current study, that the extant WHL populations have low sequence diversity in the mtDNA control region. mtDNA variability in the WHL is markedly less than that of several other endangered primate species (Table 1). Specifically, the HVRI haplotype diversity of our WHL samples was only half of the values of most of those species and its nucleotide diversity was a magnitude lower than that of those species. This result agrees with our previous study that showed fewer numbers of alleles per locus and lower heterozygosity levels of microsatellite markers in the WHL, compared to those of the Guizhou and Yunnan snub-nosed monkeys [22].

**Table 1:** Comparison of population genetic parameters inferred from microsatellite data from 6 endangered primate species. *L*, sequence length*; n*, number of haplotypes; *s*, number of polymorphic sites; *h*, haplotype diversity; *π*, nucleotide diversity; SD, standard deviation; CR, critically endangered; EN, endangered

Species	*Trachypithecus leucocephalus*	*Trachypithcus francoisi*	*Rhinopithecus brelichi*	*Brachyteles hypoxanthus*	*Ateles geoffroyi*	*Rhinopithecus bieti*
Common name	White-headed langur	François’s langur	Guizhou snub-nosed monkey	Northern muriqui	Black-handed spider monkey	Yunnan snub-nosed monkey
Endangered status	CR	EN	EN	CR	EN	EN
Population size	1000	2000^a^	750–800	864	870–924	2000
Sample size	213	178	141	152	162	157
*L*	350	395	379	366	221	379
*n*	9	29	5	23	36	30
*s*	10	Unspecified	25	21	31	51
*h* (SD)	0.486 (0.036)	0.952 (0.005)	0.457 (0.048)	0.905	0.880	0.945 (0.006)
*π* (SD)	0.003 (0.000)	0.034 (0.001)	0.014 (0.007)	0.014	0.014	0.036 (0.018)
References	This study	[63]	[41]	[40]	[64]	[41]

^a^Population size in China [65]

Low genetic diversity in natural populations can result from a number of different evolutionary and demographic processes. Using several population demographic inference methods, we detected no genetic signature of historical population declines (via analyses of mtDNA) or recent demographic bottlenecks (via analyses of microsatellite data) in the FS, CZ, or the population as a whole. However, the population assumptions and detection powers of these methods vary considerably, and potential confounding factors are present in sampling schemes and population genetic characteristics that can lead to false signals [66]. For example, Bayesian skyline plots are prone to the confounding effect of population structure with population size declines [67]. This effect is most prominent when all samples are collected within a deme, and least influential when sampling is balanced (i.e., multiple samples from each deme) [68]. Our sampling strategy maximized sampling coverage for the extant populations, and we conducted the Bayesian skyline plot analyses on both a local (FS) and the entire population. We think that these sampling and analytical strategies diminished the effect of population structure on our demographic inference. In addition, we used multiple methods (i.e., Fu’s *F*_s_, Tajima’s *D*, mismatch distribution, and Bayesian skyline plot analyses of mtDNA; and BOTTLENECK and DIYABC analyses of microsatellites) to evaluate population size changes. The overall consistent patterns yielded from these analyses support a constant historical population with no large, long- or short-term size fluctuations. Therefore, the observed low genetic diversity in this species is unlikely to be explained mainly by loss of historical genetic diversity. Two underlying mechanisms may account for the low genetic diversity in the contemporary populations. First, the shallow evolutionary history of the species may have restricted the accumulation of population genetic variations. Mitochondrial sequence analysis showed that the WHL forms a monophyletic clade within Francǫis’s langurs (*Trachypithecus francoisi*) and that it split from the latter 0.46–0.27 million years ago [63]. These 2 species currently inhabit adjacent ranges separated by rivers and other geographic barriers and seem to represent a typical case of peripatric speciation [69]. The inferred divergence time is a rather short evolutionary timeframe in primates, considering their long generation time. In line with their short divergence history, crossing experiments have shown that the 2 species could interbreed and produce fertile offspring [70]. This indicates the lack of a physiological reproductive barrier between the 2 species, suggesting limited interspecific genetic differentiation and, possibly, intraspecific diversification in the WHL post divergence. Second, the relatively small historical population size, confined to suitable habitat, may have restricted population genetic diversity. WHL distribution is restricted by geographic barriers (i.e., rivers, mountains, and large flatlands) and the species’ suitable habitat, limestone forests, is only found in a small fraction of its already limited range. Based on the mtDNA skyline analysis, using a female generation time of 10 years, we estimated a historical female effective population size of 1000–3000 individuals. Assuming an equal sex ratio, the total effective population will be in the range of 2000–6000 individuals, which is close to the maximum historical population of 5000, an estimate based on both the total suitable habitat area (360 km^2^) and on a recent population density census at CZ (13.9 langurs/km^2^; [26]). The total historical effective population size of FS and CZ, inferred by DIYABC simulations, was 3120 (95% CI: 1757–4760), a match with the aforementioned historical population size estimates. Taken together, these analyses suggest that the WHL had a small historical population size. As genetic diversity of selectively neutral genes is proportional to effective population size [1], the small historical population, restricted by suitable habitat range, most likely limited the WHL from attaining greater genetic diversity.

### Divergence history of the FS and CZ populations

Little is known about the evolutionary divergence history of the extant WHL populations. The FS population showed considerably greater mtDNA genetic diversity than did the CZ population, as the latter possesses only 1 haplotype that is also the most common haplotype of the FS population, suggesting that the FS population may be the source from which the CZ population has recently descended. Alternatively, the CZ population may have been established early but lost genetic variation during recent, severe demographic bottlenecks caused by human-related habitat destruction and hunting [24]. However, DIYABC analysis results support the first hypothesis. Interestingly, the 82 CZ individuals showed only 1 haplotype in their mtDNA HVRI sequences, yet genetic diversity (numbers of alleles, allelic richness, and observed heterozygosity) of the CZ population microsatellite markers was similar to that in the FS population [22]. This pattern suggests that the CZ population was established by very few females, hence the low variation in maternally inherited mtDNA, but then a greater number of founder males boosted genetic diversity of bi-parentally transmitted markers (i.e., nuclear microsatellites). This speculation makes sense, given the WHL reproductive system, which is characterized by strongly male-biased dispersal. Males can migrate long distances whereas females often remain in or near their natal groups [31, 38, 71]. Despite apparent WHL population declines across its distribution ranges during the later decades of the last century, our BOTTLENECK tests and DIYABC simulations provided no support for the occurrence of recent population bottlenecks. However, it is common for microsatellite-based bottleneck tests to fail to detect bottlenecks in populations known to have experienced recent declines (reviewed in [72]). Several factors, including the scale of population declines, timeframe of the declines and recovery, and pre-bottleneck genetic diversity, can influence and confound the detection of population bottlenecks [73, 74]. Detection of population declines by BOTTLENECK tests is most effective when the proportional declines are very large (e.g., 10–1000-fold) or post-bottleneck population size is very small [72]. The negative results of our BOTTLENECK tests may be attributed to limited demographic reductions and/or relatively low population genetic diversity pre-bottleneck. Inclusion of potential museum samples from earlier time periods would lend better evidence for population diversity changes incurred by recent population declines. Recently, the ABC method has provided a powerful and versatile approach for inferring population demographic history and has gained popularity in population genetics [75, 76]. However, contemporary genetic patterns in natural populations are the products of complex interactions between various evolutionary and anthropogenic processes, the details of which cannot be fully captured by statistical inferences. Simulation-based evaluation of the ABC approach has shown increased rates of erroneous inferences when candidate models are many, similar, or complex [77]. In this study, we focused on 4 scenarios of population demography, including the most influential demographic events (i.e., population divergence and a population bottleneck). This allowed for a greater probability of identifying either the true model or the one most like it. In summary, the WHL’s population history is undoubtedly more complex and dynamic than the evaluated demographic scenarios and this must be considered when interpreting the results of ABC-based inferences.

### Conservation implications

Although our results suggest that the WHL’s low genetic diversity may be due primarily to its short evolutionary history and its small historical population size, recent anthropogenic range contractions and population declines have possibly caused further reductions in WHL effective population size and genetic variation, thus potentially aggravating the effects of inbreeding and genetic depression. Depletion of genetic variability in wild populations has been linked to reduced reproduction and to lowered resistance to infectious diseases, both of which threaten the fitness and long-term survival of populations [3]. Although we have not found evidence of phenotypic defects associated with inbreeding depression (e.g., reduced birth and infant survival rates, increased susceptibility to diseases, etc.) in the WHL populations, small effective population size and extended genetic isolation can pose serious threats for the long-term survival of affected endangered species [78]. Time-lag effects of habitat modification on population structure and genetic isolation, especially in species with long life cycles, may not be fully captured by these analyses. Also, the negative impacts of genetic inbreeding on population fitness and adaptive potential can exacerbate with time. Hence, recovery of both habitat area and connectivity through the restoration of migration corridors between WHL habitat fragments should be a conservation priority. Such actions would enhance inter-population gene flow and combat the effects of small population size. We also suggest continuous and close monitoring of the population’s reproductive performance and health conditions to ensure sustainable conservation of the WHL.

## Conclusions

Our study was the first to apply both mtDNA and nuclear microsatellite markers to a large non-invasive sample of wild WHLs to evaluate the long- and short-term population demographic history of this critically endangered primate. Analysis of mtDNA sequence data revealed extremely low population genetic diversity relative to other endangered primates and a lack of genetic variation in the CZ population. The demographic history, revealed by mtDNA and microsatellites, indicated a small and stable historical effective population size and no recent population bottlenecks. In addition, population demographic history simulations generated by DIYABC analyses supported the hypothesis that the CZ population had recently been established by founders from the FS population. These results suggest that shallow evolutionary history and small historical population size are the primary factors shaping the WHL’s contemporary genetic diversity. Priority must be given to habitat area recovery and the restoration of habitat connectivity in order to combat the potentially detrimental effects of genetic inbreeding in the remaining, small WHL populations.

## Methods

### Study area and species

The WHL is endemic to an area bound by rivers and mountains in Guangxi Province, southwest China, and has an estimated historical range of about 360 km^2^ [21]. It is an arboreal, folivorous colobine primate adapted to karst limestone habitat [29]. Its social system is characterized by 1-male, multi-female (range 1–14) reproductive groups with male dispersal and female philopatry [30]. Reproductive groups have relatively stable home ranges and males exhibit strong territorial behavior [31].

This species was first described scientifically in 1955 and named in 1957 [32], and no data are available regarding its historical population status before the 1980s. Large range contractions and population declines were recorded in the last 2 decades of the twentieth century [21, 23, 33]. Population declines have ended since both the establishment of nature reserves covering most of the WHL’s range and the implementation of a strict ban on hunting in the 1980s. The most recent range-wide population census reported a total population size of about 1000 (FS: about 550 langurs in 60–70 reproductive groups; CZ: about 300 langurs in 30–40 reproductive groups) and an overall trend of increasing population size since the early 2000s [26].

Average female reproductive age are 5–6 years at first birth [34] and about 20 years at last birth (W. Pan, personal observations). Because the generation times of great apes are close to the first one-third of their reproductive lives [35], we estimated the generation time for female WHLs to be around 10 years. WHL males begin reproducing a few years later than females, possibly because they can only invade and become the resident male of a reproductive group when they have reached full adult size. In one documented case, a male became the resident male at 8 years of age (W. Pan, personal observations). Male residency lasts an average of 50 months [36]; therefore, 10 years is a reasonable estimate for male generation time.

## Additional file

Additional file 1: **Table S1.** Primer information for PCR amplifications of 15 microsatellite markers in the white-headed langur. **Table S2.** Primer information for PCR amplifications of the white-headed langur mtDNA control region sequences and sex identification marker. **Table S3.** Prior values of parameters for simulated scenarios in DIYABC analysis. **Table S4.** Polymorphic sites in the mtDNA HVRI sequences and the number and sampling locations of the haplotypes. **Table S5.** Parameters of genetic diversity and results of neutrality tests using the mtDNA HVRI sequences in the Fusui (FS) and Chongzuo (CZ) populations. **Table S6.** Summary of BOTTLENECK analyses based on the microsatellite data from the Fusui (FS) and Chongzuo (CZ) populations. **Table S7.** Summary of posterior probabilities of 4 demographic history scenarios evaluated in DIYABC analysis using microsatellite data. **Table S8.** Posterior distributions of population demographic parameters from the scenario with the highest posterior probability (Scenario 2) inferred by DIYABC analysis using microsatellite data. **Figure S1.** Model checking by applying a PCA on the best-supported scenario (Scenario 2) in DIYABC analysis. **Figure S2.** Point estimates of effective population sizes (N1 and N2) and the temporal parameter (t1) of the best supported scenario (Scenario 2) from DIYABC analysis. (PDF 374 kb)

## Data Availability

Sequences of mtDNA HVRI haplotypes are available in GenBank (Accession nos. KP772243–KP772251). Microsatellite data are available in the Dryad Digital Repository (doi:10.5061/dryad.746 k3).

## Abbreviations

CZ: Chongzuo
FS: Fusui
HVRI: hypervariable region I
mtDNA: mitochondrial DNA
PCA: principal component analysis
SMM: stepwise mutational model
TPM: two-phased stepwise mutational model
WHL: white-headed langur
