# Supplementary material for: Low genetic diversity in a critically endangered primate: shallow evolutionary history or recent population bottleneck?
Source: BMC Evol Biol. 2019 Jun 26;19:134. doi: 10.1186/s12862-019-1451-y (PMC6595580; doi:10.1186/s12862-019-1451-y)
Supplement: Supplementary file 1 — Table S1. Primer information for PCR amplifications of 15 microsatellite markers in the white-headed langur. Table S2. Primer information for PCR amplifications of the white-headed langur mtDNA control region sequences and sex identification marker. Table S3. Prior values of parameters for simulated scenarios in DIYABC analysis. Table S4. Polymorphic sites in the mtDNA HVRI sequences and the number and sampling locations of the haplotypes. Table S5. Parameters of genetic diversity and results of neutrality tests using the mtDNA HVRI sequences in the Fusui (FS) and Chongzuo (CZ) populations. Table S6. Summary of BOTTLENECK analyses based on the microsatellite data from the Fusui (FS) and Chongzuo (CZ) populations. Table S7. Summary of posterior probabilities of 4 demographic history scenarios evaluated in DIYABC analysis using microsatellite data. Table S8. Posterior distributions of population demographic parameters from the scenario with the highest posterior probability (Scenario 2) inferred by DIYABC analysis using microsatellite data. Figure S1. Model checking by applying a PCA on the best-supported scenario (Scenario 2) in DIYABC analysis. Figure S2. Point estimates of effective population sizes (N1 and N2) and the temporal parameter (t1) of the best supported scenario (Scenario 2) from DIYABC analysis. (PDF 374 kb) [file 12862_2019_1451_MOESM1_ESM.pdf]

Additional Files for:

Low genetic diversity in a critically endangered primate: shallow evolutionary history or recent population bottleneck?

Weiran Wang[1,2,3], Yitao Zheng[1,2], Jindong Zhao[1,2], Meng Yao[1,2*]

[1]School of Life Sciences, Peking University, Beijing 100871, China

[2]Institute of Ecology, Peking University, Beijing 100871, China

[3]Beijing National Day School, Beijing 100871, China

Table S1 Primer information for PCR amplifications of 15 microsatellite markers in the white-headed langur [1]. T_a, annealing temperature.

**Locus**	**Size range (bp)**	**Repeat structure**	T_a **(°C)**	**Primer sequences (5′-3′)**	**Dye label**
WHL-60	141–149	$(AGAT)_{10}$	54–60	F-CAGTTAAGGCTCCTTCAGA<br>R-GCCAACAGCTTCACAT	HEX
WHL-121	153–157	$(ATAG)_5$	55–60	F-AAAGAGGAAAGGCAGGTA<br>R-CAAAGCCAGACTGTCTCAA	HEX
WHL-122	105–111	$(TG)_{14}$	53–60	F-CTGCCTGAGCTACATAGTGA<br>R-AACATCCTTGGCTCTTGG	FAM
WHL-138	206–218	$(GATA)_{10}$	53–60	F-CTGTTCCATAAGCTGAGGTA<br>R-GACGATAGCAATTCTGTGAG	HEX
WHL-206	201–213	$(AC)_{21}$	55–58	F-GCACATTTATAGCAGCACA<br>R-ATAGTCCCTAACTCCATCC	FAM
WHL-220	179–189	$(GT)_{20}$	48–56	F-CCTTCTCATCAACAAATCC<br>R-AAACCTCAATCTATCACTCA	FAM
WHL-287	166–179	$(AC)_{16}$	48–59	F-TGCCACGATATGTTGGAGA<br>R-GCTTCTTAGGCGTAGTTTC	TAMRA
WHL-293	185–192	$(GT)_{19}$	49–53	F-TGAGGAAGTGAAATGAA<br>R-GAAGTAGTAACAGTGGAGT	HEX
WT-58	104–114	$(AC)_{12}$	44–57	F-AAAGTTCTACGCTGTTGTT<br>R-TGAGATGGAATGTGATTGT	TAMRA
WT-97	150–154	$(CA)_8(AC)_7$<br>$(AC)_7$	57–60	F-ATGCCAAGCCTTGTGCTG<br>R-CTGCCTTCAATAGATGTCCTACCT	HEX
WT-111	171–183	$(AC)_{13}$	53–60	F-GAGCCCATAGAAGGTAGT<br>R-GGTTGTTGACATCATCCC	HEX
WT-168	210–222	$(TAGA)_7$	58–60	F-CTTATCATTTGTTGCGTGTC<br>R-ATCTTCCAGGCTGCTCTT	FAM
WT-173	163–182	$(TG)_{21}$	48–60	F-TTTTCCTTCTACCACCTCA<br>R-TTCACTTCATCTCAACCCT	HEX
WT-181	124–144	$(TCTA)_{11}$<br>$(TG)_{13}$	55–57	F-TGCAGGGAGTCTAACAGT<br>R-CACATTCCTTGGGCTTTT	TAMRA
WT-223	169–175	$(AC)_5(CA)_8$	45–56	F-ATAAGTGATTTAGGGCAAT'<br>R-TTTCCTATGAGCACAAGAT	HEX

Table S2 Primer information for PCR amplifications of the white-headed langur mtDNA control region sequences and sex identification marker. CR, control region; HVRI and HVRII, hypervariable region I and II; T_a, annealing temperature.

Primer	Target fragment	Size (bp)	T_a (°C)	Primer sequences (5′-3′)	Reference
**mtDNA**					
**P3-P4**	CR HVRI	395	55	F: AACTGGCATTCTATTTAAACTAC<br>R: ATTGATTTCACGGAGGATGGT	[2]
**P3-P470**	CR HVRI	574	55	F: AACTGGCATTCTATTTAAACTAC<br>R: TGACTGGTTAATAGGGTGATAG	[2]
**P78-P79**	CR central domain	427	55	F: CAACTTAATGTCTTCATTATCG<br>R: ACGTAGGTGCGGTTAATGAT	[2]
**P82-P87**	CR HVRII	607	55	F: TAACCAGTCACGGGAGCTCT<br>R: GGGGATGCTTGCATGTGTAA	[2]
**Sex marker**					
**Deadbox**	DEAD-box gene	X:178<br>Y:208	62	F:TGATGTTTAGTGCTACTTTTCCTAAGGAA<br>R:AGAGGTAGAGCCWACTCTKCCTACA	[3]

Table S3 Prior values of parameters for simulated scenarios in DIYABC analysis. A uniform distribution was applied for all parameters. N_1, FS effective population size; N_2, CZ effective population size after divergence or after the proposed bottleneck; N_{2a}, CZ effective population size before the proposed bottleneck (Scenario 4).

Parameter	Minimum	Maximum
Effective population size		
N_1	100	3500
N_2	100	1500
N_{2a}	100	3000
Time scale in generations		
t_1	10	400
t_2	10	1500
Mutation model		
Mean mutation rate	1×10^{-5}	1×10^{-3}
Individual locus mutation rate	1×10^{-5}	1×10^{-3}

Table S4 Polymorphic sites in the mtDNA HVRI sequences and the number and sampling locations of the haplotypes of the white-headed langur. Vertical numbers indicate polymorphic sites in the 350 bp fragment. Blanks in sequence alignment represent identical nucleotides as Hap A. Hap H and Hap I co-occurred in 5 samples and were counted only once in total numbers. FS, Fusui; CZ, Chongzuo.

	**Polymorphic sites**												
						**1**	**1**	**1**	**1**	**3**			
		**3**	**4**	**4**	**5**	**1**	**2**	**2**	**5**	**2**			**Total**
**Haplotype**	**9**	**6**	**6**	**8**	**1**	**8**	**1**	**7**	**2**	**8**	**FS**	**CZ**	**population**
Hap A	C	G	T	T	G	A	T	T	C	T	41	0	41
Hap B				C							8	0	8
Hap C				C	A						65	82	147
Hap D	G			C	A						1	0	1
Hap E				C	A		C				1	0	1
Hap F		A		C	A						3	0	3
Hap G				C	A	G		C		C	7	0	7
Hap H			C	C	A				T		5	0	5
Hap I				C	A				T		5	0	5
**Total**											131	82	213

Table S5 Parameters of genetic diversity and results of neutrality tests using the mtDNA HVRI sequences in the Fusui (FS) and Chongzuo (CZ) populations. *N*, number of individuals; *n*, number of haplotypes; *s*, number of polymorphic sites; *h*, haplotype diversity; *π*, nucleotide diversity. SD, standard deviation.

**Population**	***N***	***n***	***s***	***h* (SD)**	***π* (SD)**	**Tajima's *D* (*P*)**	**Fu's F_s (*P*)**
**FS**	131	9	10	0.653	0.0040	−0.576	−1.197
				(0.030)	(0.0003)	(0.321)	(0.333)
**CZ**	82	1	0	0	0	—	—
**Total**	213	9	10	0.486	0.0028	−0.968	−2.153
				(0.036)	(0.0003)	(0.184)	(0.204)

Table S6 Summary of BOTTLENECK analyses based on the microsatellite data from the Fusui (FS) and Chongzuo (CZ) populations.

	Sign test *P*-value		Wilcoxon signed-rank test *P*-value		
**Population**	SMM	TPM	SMM	TPM	**Mode-Shift**
**FS**	0.290	0.503	0.300	0.084	L-shaped
**CZ**	0.327	0.152	0.227	0.154	L-shaped
**Total**	0.255	0.266	0.756	0.445	L-shaped

Table S7 Summary of posterior probabilities of 4 demographic history scenarios evaluated in DIYABC analysis using microsatellite data.

**Scenarios**	**1**	**2**	**3**	**4**
Posterior probabilities	0.0293	0.4599	0.2518	0.2590
Confidence intervals	[0.0225-0.0362]	[0.4469-0.4729]	[0.2400-0.2635]	[0.2471-0.2709]

Table S8 Posterior distributions of population demographic parameters from the scenario with the highest posterior probability (Scenario 2) inferred by DIYABC analysis using microsatellite data.

**Symbol**	**Median**	**0.025 quantile**	**0.975 quantile**
Effective population size			
N_1	1.98e+03	1.13e+03	3.29e+03
N_2	1.14e+03	6.27e+02	1.47e+03
Time scale in generations			
t_1	3.05e+02	1.73e+02	3.94e+02

Figure S1 Model checking by applying a PCA on the best-supported scenario (Scenario 2) in DIYABC analysis. Summary statistics, based on 5,000 simulations, visualize the fit between the simulated and the observed datasets.

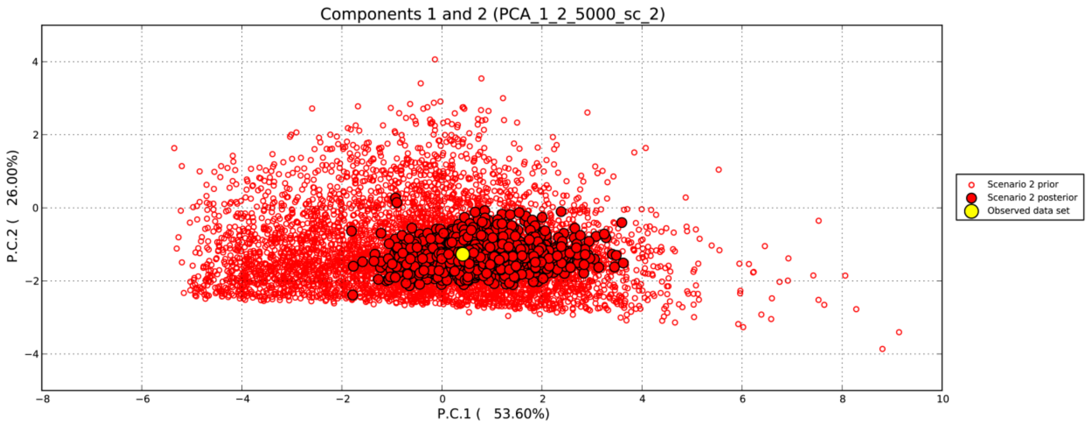

Figure S2 Point estimates of effective population sizes (N1 and N2) and the temporal parameter (t1) by local linear regression on 1% of the simulated dataset closest to the observed dataset of the best supported scenario (Scenario 2) from DIYABC analysis. The *x*-axis indicates time in generations and the *y*-axis shows the marginal probability.

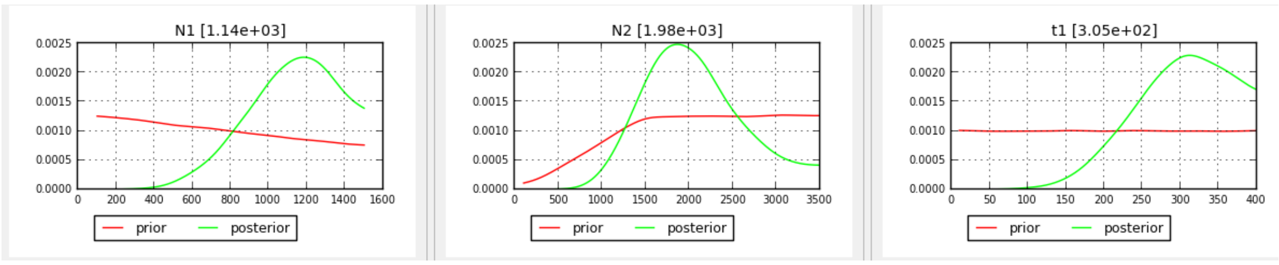

References

1. Wang W, Qiao Y, Zheng Y, Yao M. Isolation of microsatellite loci and reliable genotyping using noninvasive samples for a critically endangered primate, *Trachypithecus leucocephalus*. Integr Zool. 2016;11:250-62.
2. Wang W, Qiao Y, Pan W, Yao M. Low genetic diversity and strong geographical structure of the Critically Endangered white-headed langur (*Trachypithecus leucocephalus*) inferred from mitochondrial DNA control region sequences. PLoS ONE. 2015;10(6):e0129782.
3. Villesen P, Fredsted T. A new sex identification tool: One primer pair can reliably sex ape and monkey DNA samples. Conserv Genet. 2006;7:455-9.
